# Morbidity Pattern Among Adult Patients at the National Health Insurance Scheme Clinic of a Tertiary Hospital, Southwest Nigeria

**DOI:** 10.7759/cureus.37529

**Published:** 2023-04-13

**Authors:** Olusoji A Solomon, Demilade O Ibirongbe, Oluremi O Solomon

**Affiliations:** 1 Department of Family Medicine, Faculty of Clinical Sciences, Ekiti State University, Ado Ekiti, NGA; 2 Department of Community Medicine, University of Medical Sciences (UNIMED), Ondo, NGA; 3 Department of Community Medicine, Faculty of Clinical Sciences, Ekiti State University, Ado Ekiti, NGA

**Keywords:** nigeria, nhis, disease pattern, mortality, morbidity

## Abstract

Background: For adequate planning of healthcare services, a good knowledge of the burden and pattern of morbidity and mortality in the community is a key requirement. This study aimed at describing the morbidity pattern among patients at a National Health Insurance Scheme (NHIS) clinic in Southwestern Nigeria.

Method: This was a cross-sectional study. Secondary data was extracted from case notes of 5,108 patients who visited the NHIS Clinic in a tertiary health facility in Southwestern Nigeria, from 2014 to 2018, using the International Classification of Primary Care (ICPC-2) to categorize the diseases. Data analysis was done using IBM SPSS Statistics for Windows, Version 25.0 (Released 2018; IBM Corp., Armonk, NY, USA).

Result: Females were 2,741 (53.7%), males were 2367 (46.3%), and the mean age was 36.7±9.5 years. General and unspecified diseases were the commonest presentations. Malaria (1,268; 45.5%) was the commonest disease among the patients. Sex and age were associated with disease distribution (p-value = 0.001).

Conclusion: Public health preventive strategies and measures should be undertaken to address the priority diseases as shown in this study.

## Introduction

The knowledge of the distribution, pattern, and burden of diseases in a community is very important for planning and setting up policies in public health services. Facility-based data is a good alternative in providing data on the morbidity profile of a particular community especially when community-based data are not readily available.

Earlier surveys showed the burden of communicable diseases to be higher than non-communicable diseases (NCDs); however, NCDs are increasing worldwide with their consequent complications and increased morbidity especially in most developing countries [1,2]. Reports have shown that greater than 40 million people die annually from NCDs which account for 70% of global deaths; out of this, more than 15 million die at a younger age [3]. Premature death also accounts for greater than 80% of the burden in low- and middle-income countries [4,5].

The National Health Insurance Scheme (NHIS) of Nigeria is a social health security system in which the healthcare of an employee is paid for by both the employee and the employer [6]. The main objective of NHIS is to achieve equitable access to healthcare in Nigeria toward universal health coverage [6]. It is also an alternative source of funding for the increasingly costly healthcare system [6]. Little research has been done on the morbidity pattern of patients accessing care at NHIS-accredited health facilities in Nigeria, providing such evidence-based reports in these facilities will aid in planning health services efficiently with the consequent provision of enhanced quality services to the community [3]. This study sought to answer these questions: What is the morbidity pattern in the NHIS Clinic of Ekiti State University Teaching Hospital (EKSUTH), Ado-Ekiti, Nigeria? Is there any association between age, sex, and the morbidity pattern in the NHIS Clinic of Ekiti State University Teaching Hospital, Ado-Ekiti, Nigeria? This study therefore aimed at describing the morbidity pattern among patients who presented from 2014 to 2018 at the NHIS clinic of a Teaching Hospital in the Southwest of Nigeria and the association between sex, age, and the morbidity pattern.

## Materials and methods

The study was conducted at the National Health Insurance Scheme (NHIS) Unit of the Family Medicine Department, Ekiti State University Teaching Hospital (EKSUTH), Ado Ekiti, Southwest Nigeria. The hospital is a tertiary hospital that serves people from Ekiti, Ondo, and Kwara states and its environs. The unit is led by one consultant and has five medical officers. There are 12 nursing officers who run an eight-hour shift with a minimum of three nurses per shift and are assisted by two health assistants and a porter. In the NHIS Clinic of this hospital, all cases are seen in primary care, while in secondary care, selective cases are seen according to the operational guideline.

This is a cross-sectional study covering the duration of five years from January 2014 to December 2018. The number of newly presented patients aged 20 years and above during each clinic day covering this period was collated from the NHIS register, and their records were retrieved to extract relevant information about their first encounter and diagnosis at the NHIS clinic. The relevant information was extracted by the research assistant who was trained in this regard. The information includes patients’ biodata, clinical presentations, initial diagnosis, investigations, final diagnosis, and relevant clinical outcomes. This data was entered into a predesigned data collection sheet.

International Classification of Primary Care (ICPC-2) [7] was used to categorize the diseases. Analysis was done by using IBM SPSS Statistics for Windows, Version 25.0 (Released 2018; IBM Corp., Armonk, NY, USA). A descriptive analysis of the sociodemographic characteristics of the respondents and the morbidity pattern of diseases was carried out using tables and percentages. Inferential statistics was done using chi-square to check the association between disease classification and some sociodemographic characteristics (age and sex). A significant p-value was set at < 0.05. The approval for this study was obtained from the Ethical Review Committee of the Ekiti State University Teaching Hospital (ethical approval number: EKSUTH/A67/2020/05/008).

## Results

The total number of patients who presented in the NHIS Clinic aged 20 years and above between 2014 and 2018 was 5,108. Table 1 shows that patients between the ages of 20 and 39 years were 69.2%, while the mean age was 36.7 ± 9.5 years. Females were 53.7%, and most of the patients (95.9%) were Christians. Four thousand five hundred and eight (88.3%) of them had tertiary education, 4,719 (92.4%) were married, and 242 (4.7%) resides in rural areas of Ekiti State.

**Table 1:** Sociodemographic Characteristics of Patients Attending NHIS Clinic NHIS: National Health Insurance Scheme.

Age	Frequency (n = 5108)	Percentage
20-29	1050	20.6
30-39	2480	48.6
40-49	1043	20.4
50-59	401	7.9
60-69	87	1.7
70 and above	47	0.9
Gender		
Male	2367	46.3
Female	2741	53.7
Religion		
Christian	4899	95.9
Muslim	209	4.1
Ethnicity		
Yoruba	4609	90.2
Igbo	152	3.0
Hausa	10	0.2
Others	337	6.6
Educational level		
Primary	19	0.4
Secondary	581	11.4
Tertiary	4508	88.3
Marital status		
Married	4719	92.4
Single	389	7.6
Occupation		
Student	454	8.9
Civil/Public servant	3730	73.0
Business	460	9.0
Others	464	9.1
Place of residence		
Urban	4866	95.3
Rural	242	4.7

Out of 5,108 patients attended, 1,435 (28.1%) came with diseases or symptoms classified under general and unspecified, 514 (10.1%) under musculoskeletal, 460 (9.0%) under eye, 623 (12.2%) under digestive, 359 (7.0%) under respiratory, and 66 (1.3%) under neurological as shown in Table 2.

**Table 2:** International Classification of Primary Care Diseases Presented in NHIS Clinic ICPC: International Classification of Primary Care, NHIS: National Health Insurance Scheme.

Serial no	ICPC Code	Frequency (n = 5108)	Percentage
1	General and unspecified	1435	28.1
2	Blood, blood-forming organs, and immune mechanism	26	0.5
3	Digestive	623	12.2
4	Eye	460	9.0
5	Ear	60	1.2
6	Cardiovascular	241	4.7
7	Musculoskeletal	514	10.1
8	Neurological	66	1.3
9	Psychological	53	1.0
10	Respiratory	359	7.0
11	Skin	157	3.1
12	Endocrine/metabolic and nutritional	55	1.1
13	Urological	63	1.2
14	Pregnancy, childbearing, family planning	731	14.3
15	Female genital	189	3.7
16	Male genital	71	1.4
	Missing	5	0.1

**Figure 1:**
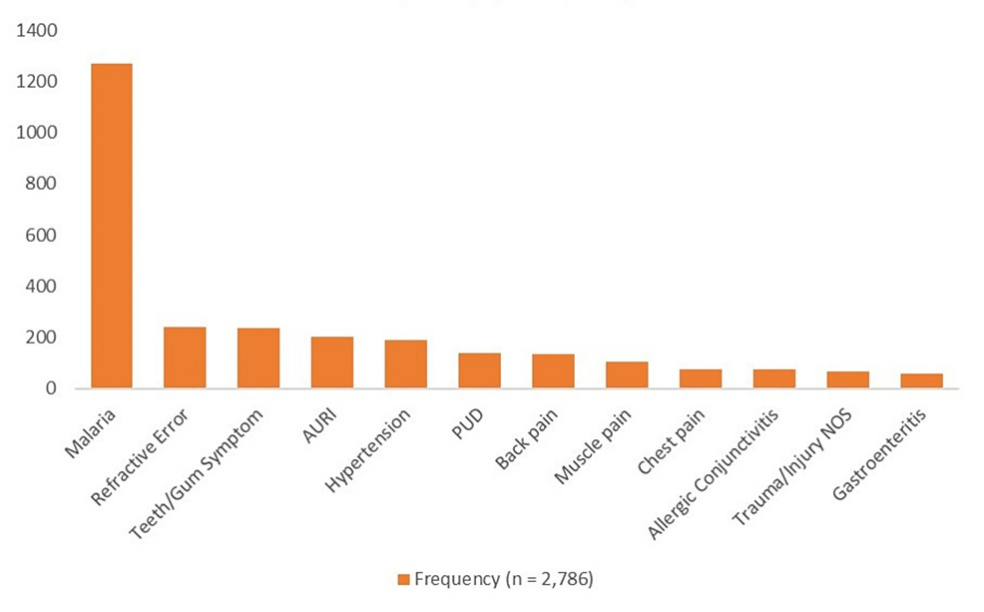
Morbidity Pattern of 10 Common Diseases Among the Patients AURI: acute upper respiratory infection, PUD: peptic ulcer disease, NOS: not otherwise specified.

Figure 1 shows that out of the 12 commonest diseases among the patients, malaria (1,268; 45.5%) was the highest, followed by the refractive error of 241 (8.7%). Hypertension among the respondents was 188 (6.7%) and peptic ulcer disease was 139 (5.0%), while gastroenteritis was 57 (2.0%).

The distribution of the diseases along the age group as shown in Table 3 revealed that ages between 20 and 45 had a higher proportion compared to those above 45 years of age. This difference is statistically significant with a p-value of 0.001. The distribution of the diseases between sex also revealed that the burden was generally higher among the males compared with the females; this difference is statistically significant with a p-value of 0.001.

**Table 3:** Association Between Disease Classification, Age, and Sex

ICPC Code	Age		Sex n (%)	
	20-45	>45	Male	Female
General and unspecified	1247 (87.0)	187 (13.0)	754 (52.5)	681 (47.5)
Blood, blood-forming organs, and immune mechanism	25 (96.2)	1 (3.8)	13 (50.0)	13 (50.0)
Digestive	534 (85.9)	88 (14.1)	335 (53.8)	288 (46.2)
Eye	344 (74.8)	116 (25.2)	240 (52.2)	220 (47.8)
Ear	54 (90.0)	6 (10.0)	35 (58.3)	25 (41.7)
Cardiovascular	140 (58.1)	101 (41.9)	141 (58.5)	100 (41.5)
Musculoskeletal	396 (77.0)	118 (23.0)	337 (65.6)	177 (34.4)
Neurological	45 (68.2)	21 (31.8)	38 (57.6)	28 (42.4)
Psychological	43 (81.1)	10 (18.9)	31 (58.5)	22 (41.5)
Respiratory	307 (85.5)	52 (14.5)	189 (52.6)	170 (47.4)
Skin	137 (87.3)	20 (12.7)	95 (60.5)	62 (39.5)
Endocrine/metabolic and nutritional	30 (54.5)	25 (45.5)	25 (45.5)	30 (54.5)
Urological	57 (90.5)	6 (9.5)	38 (60.3)	25 (39.7)
Pregnancy, childbearing, family planning	726 (99.3)	5 (0.7)	20 (2.7)	711 (97.3)
Female genital	171 (90.5)	18 (9.5)	5 (2.6)	184 (97.4)
Male genital	53 (74.6)	18 (25.4)	70 (98.6)	1 (1.4)
Statistical indices	381.758	p-value = 0.001	948.514	p-value = 0.001

## Discussion

The present study was carried out to observe morbidity patterns among the participants. It revealed that the total number of patients who presented at the National Health Insurance (NHIS) Clinic aged 20 years and above between 2014 and 2018 was 5,108, giving an average annual patient load of 1,021. Young adults (69.2%) constituted most of the patients who were seen at the NHIS Clinic during the period under review, with the mean age being 36.7 ± 9.5 years; this is reflective of Nigeria's young population.

A majority of the patients have tertiary education, work as civil/public servants, and reside in urban centers. This may not be unconnected to the demography that characterizes the area where the health facility is located. These people will have more access to NHIS than those residing in rural areas and who have lower levels of education or are unemployed/self-employed. The slightly higher proportion of females (53.7%) is consistent with other similar studies [8-12] and may be because women show more anxiety about their health and are more predisposed to seeking care earlier and quicker. With the benefit of this hindsight, priority should be given to planning health services for the female gender.

Based on the International Classification of Primary Care Diseases which we used to group the diseases recorded among patients who presented at the Clinic, the most frequent presentation was those with diseases or symptoms classified under general and unspecified (28.1%), closely followed by pregnancy/childbearing/family planning (14.3%), and digestive (12.2%). Our finding is similar to that of a survey of a predominantly female population involving children and adults, in Lagos, Nigeria, which found the top five ICPC-2 classes were general unspecified, pregnancy/childbearing/family planning, respiratory, digestive, and musculoskeletal, while social problems have the least frequency [10]. However, digestive, eye, neurological, musculoskeletal, and general body symptoms were the top five diseases found in another similar study in elderly populations presenting at a Primary Care Clinic in Ibadan, Nigeria [13]. Our study differs from this possibly because we have a much younger population.

A more detailed evaluation of the commonest diseases among the patients showed that malaria fever was the highest (45.5%), followed by refractive error (8.7%). Hypertension (6.7%) and back pain (4.8%) were not significantly high since we have a young demographic; our study also showing in the distribution of the diseases along the age group that ages between 20 and 45 years had a significantly higher proportion compared to those above 45 years of age (p-value = 0.001). Malaria is endemic in Nigeria with a high public health burden, and other studies in Nigeria have reported similar findings [8,13-15]. Nigeria accounts for about 29% of the global burden of malaria. In Nigeria, malaria is responsible for approximately 60% of outpatient visits and 30% of admissions [16]. Our finding contrasts however with a study carried out in North India among patients attending an urban health center in which out of total reported cases at outpatient department (OPD), respiratory diseases (20.0%), gastrointestinal tract (GIT) diseases (8.0%), diarrheal diseases (8.0%), bone and joint diseases (8.64%), and hypertension constituted the principal cause of morbidity in this study population [3]; as well as another study in Bangladesh in which GIT disorders followed by endocrine disorders were the commonest presentations [12]. This difference is likely due to differing environmental factors and geographical in both areas, Nigeria being a tropical climate that favors the proliferation of mosquitoes and other insect vectors.

In this study, a disaggregation of the ICPC-2 pattern of disease by gender revealed that the burden was generally statistically significantly higher among the males compared with the females (except in endocrine/metabolic and nutritional, pregnancy, childbearing, family planning, and female genital chapters). This slightly differs from the study in North Central Nigeria; where in the digestive, cardiovascular, and endocrine/metabolic chapters, a significantly larger percentage of women were affected than men, while in the neurological chapter, men were more significantly affected than women [8]. Also, in contrast, a study among geriatric patients in Southwestern Nigeria discovered a significantly higher proportion of females presented with musculoskeletal problems compared to males, while likewise, more females presented with generalized symptoms such as body pains and fever compared to males [17]. The difference in this study may be because the proportion of males in the civil/public service is more, and the population using the NHIS service are predominantly civil/public servants; also of note is the age of the respondents, since these diseases are common with old age.

The findings of this study should be considered in light of limitations arising from the study being a facility-based retrospective study and secondary data being used for drawing inferences; hence generalization of our findings to the entire community needs some caution. Moreover, the study was conducted in a health insurance clinic, and the disease pattern might be different in a general clinic for uninsured persons. The study also does not explore the causes of the observed patterns.

## Conclusions

The study discussed the morbidity pattern of patients attending an NHIS clinic in Sub-Saharan Africa, for five years, which would assist healthcare providers, policymakers, and health administrators in planning, implementing, and improving the delivery of quality essential healthcare services. General and unspecified diseases were the commonest presentations, with malaria fever being the commonest diagnosis, consistent with the tropical background of our study. The study also showed that health problems were more common among male patients and those who were younger. Public health preventive strategies and measures should be undertaken to address the priority diseases, especially targeting the males and young population as shown in this study. More attention also should be given to maternal and child programs since pregnancy/childbirth/family planning was second in the frequency of disease classification.
